# Affinity Purification and Molecular Characterization of Angiotensin-Converting Enzyme (ACE)-Inhibitory Peptides from *Takifugu flavidus*

**DOI:** 10.3390/md21100522

**Published:** 2023-09-29

**Authors:** Yongchang Su, Shicheng Chen, Shuji Liu, Yin Wang, Xiaoting Chen, Min Xu, Shuilin Cai, Nan Pan, Kun Qiao, Bei Chen, Suping Yang, Zhiyu Liu

**Affiliations:** 1 College of Chemical Engineering, Huaqiao University, Xiamen 361021, China; suyongchang@stu.hqu.edu.cn; 2 Key Laboratory of Cultivation and High-Value Utilization of Marine Organisms in Fujian Province, Fisheries Research Institute of Fujian, Xiamen 361013, China; sjscut@126.com (S.L.); wangyin_83@163.com (Y.W.); xtchen@jmu.edu.cn (X.C.); xumin1315@foxmail.com (M.X.); caishuilin@hqu.edu.cn (S.C.); npan01@qub.ac.uk (N.P.); qiaokun@xmu.edu.cn (K.Q.); chenbeifjfri@foxmail.com (B.C.); 3 Medical Laboratory Sciences Program, College of Health and Human Sciences, Northern Illinois University, DeKalb, IL 60015, USA; schen1@niu.edu

**Keywords:** *Takifugu flavidus*, ACE-inhibitory activity, affinity purification, molecular docking, antihypertensive effect

## Abstract

An affinity chromatography filler of CNBr-activated Sepharose 4B-immobilized ACE was used to purify ACE-inhibitory peptides from *Takifugu flavidus* protein hydrolysate (<1 kDa). Twenty-four peptides with an average local confidence score (ALC) ≥ 80% from bounded components (eluted by 1 M NaCl) were identified by LC-MS/MS. Among them, a novel peptide, TLRFALHGME, with ACE-inhibitory activity (IC_50_ = 93.5 µmol·L^−1^) was selected. Molecular docking revealed that TLRFALHGME may interact with the active site of ACE through H-bond, hydrophobic, and electrostatic interactions. The total binding energy (ΔGbinding) of TLRFALHGME was estimated to be −82.7382 kJ·mol^−1^ by MD simulations, indicating the favorable binding of peptides with ACE. Furthermore, the binding affinity of TLRFALHGME to ACE was determined by surface plasmon resonance (SPR) with a Kd of 80.9 µmol, indicating that there was a direct molecular interaction between them. TLRFALHGME has great potential for the treatment of hypertension.

## 1. Introduction

The angiotensin-I-converting enzyme (ACE), a zinc-dependent dipeptidyl carboxypeptidase, plays a vital role in regulating blood pressure [1]. ACE catalyzes the conversion of angiotensin I (Ang I) into angiotensin II (Ang II) in the renin–angiotensin system (RAS) and degrades the vasodilating bradykinin into inactive peptides in the kallikrein–kinin system (KKS). Numerous studies showed that ACE is one of the most important therapeutic targets to control hypertension [2,3]. Therefore, ACE inhibitors have been widely applied in the clinic as effective antihypertensive drugs [4]. However, the most widely prescribed blood-pressure-lowering ACE inhibitors (captopril, enalapril, lisinopril, benazepril, and many others) were almost obtained by chemical synthesis. These drugs usually cause significant undesirable side effects, including low blood pressure, dry cough, headaches, fetal disorder, and an abnormal taste [5]. Hence, the utilization of ACE-inhibitory peptides has increasingly attracted more and more interest because they have fewer side effects and are readily prepared.

The preparation of ACE-inhibitory peptides from protein hydrolysates has always been an intense focus. ACE-inhibitory peptides can be separated using multiple techniques based on their physicochemical properties, such as molecular weight, charge difference, binding affinity, and polar nature [6]. The conventional approaches for the isolation and purification of ACE-inhibitory peptides from complex hydrolysates primarily rely on multi-stage column chromatography, including size exclusion chromatography, ion exchange chromatography, and reverse-phase high-performance liquid chromatography (RP-HPLC) [7]. However, these preparation strategies are frequently laborious and time-consuming. Moreover, some ACE-inhibitory peptides may not be detectable due to their low contents [8].

Instead, affinity chromatography is effective at enriching target-binding molecules, which has been widely used for peptide purification for decades [9]. For example, Megias et al. purified ACE-inhibitory peptides from sunflower and rapeseed protein hydrolysates by immobilizing ACE with the activated support 4 BCL glyoxyl-agarose. In the same study, they obtained inhibitory peptides with IC_50_ 50 and 150 times lower than those of the original sunflower and rapeseed hydrolysates, respectively [10]. Moreover, Sun et al. enriched and purified two novel ACE-inhibitory peptides, IVTNWDDMEL (IC_50_ = 2.08 mmol·L^−1^) and VGPAGRPG (IC_50_ = 4.66 mmol·L^−1^), from *Volutharpa ampullacea perryi* protein hydrolysate by using a combination of Zn-SBA-15-immobilized ACE and HPLC [11]. When the affinity method of immobilized ACE on magnetic metal–organic frameworks (Fe_3_O_4_@ZIF-90-ACE) was used to purify ACE-inhibitory peptides from Wakame, a novel peptide KNFL (IC_50_ = 225.87 mmol·L^−1^) was identified [12].

Computer-aided drug discovery (CADD) has become a prominent tool in drug discovery and has been widely used in drug research [13]. The application of computational approaches includes virtual screening, docking, molecular dynamics, and quantitative structure–activity relations [14]. Molecular docking and molecular dynamic simulation are widely employed techniques in molecular modeling for elucidating the structure–activity relationship of substrates. Molecular docking methodology enables the prediction of both the binding mode and affinity of small molecules within the binding site of the target protein [15]. Molecular dynamics simulations provide detailed insights into the binding process’s structural aspects by exploring the molecular mechanism further [16].

*Takifugu flavidus*, an economically significant species of pufferfish found in coastal regions of East Asia, is renowned for its delectable taste and high market value [17]. Previous studies have indicated that *T. flavidus* has the potential to function as a reservoir of biologically active peptides exhibiting ACE-inhibitory activity and antihypertensive properties [18]. In the present study, a Sepharose-immobilized ACE affinity purification method was developed for the high-efficiency enrichment of ACE-inhibitory peptides. A novel ACE-inhibitory peptide was screened from *T. flavidus,* and its interaction mechanisms with ACE were evaluated. Molecular docking and molecular dynamic simulation were performed to investigate the structural and dynamic characteristics of the peptide. Furthermore, ACE–peptide interactions were quantified using an SPR method.

## 2. Results

### 2.1. ACE-MB Enzymatic Assays

**Figure 1:**
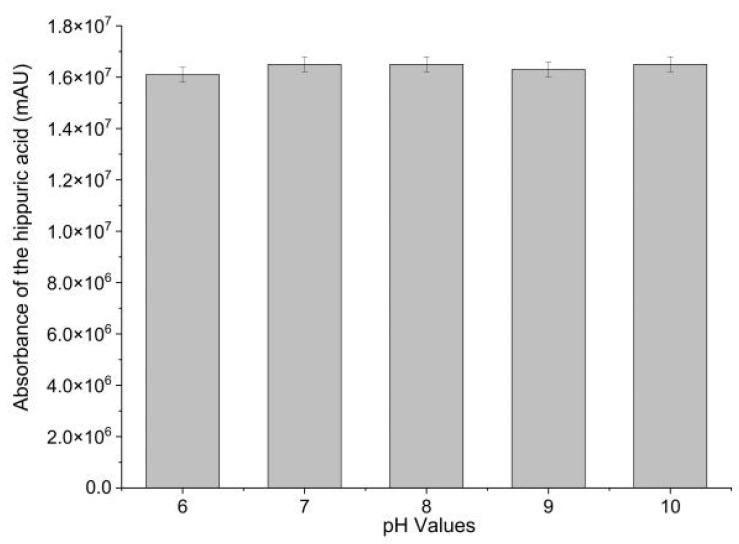
ACE-inhibitory activity of Sepharose-4B-immobilized ACE in different pHs.

Immobilization efficacy was evaluated by measuring the ACE-inhibitory activity of Sepharose-4B-immobilized ACE. ACE-inhibitory activity of Sepharose-4B-immobilized ACE was determined in different pH (6–10). Hippuric acid (HA), the reaction product, was employed as a measure of ACE-inhibitory activity. As shown in Figure 1, Sepharose-4B-immobilized ACE effectively catalyzed the production of HA from hippuryl-l-histidyl-L-leucine (HHL). This result demonstrated the successful immobilization of ACE with Sepharose 4B as well as the activity of the immobilized biomolecules. The enzyme activity was calculated to be 0.2 U·mL^−1^ immobilized enzyme. Moreover, no significant difference in ACE-inhibitory activity was observed in sodium borate buffers between pH 6.0 and 10.0, suggesting an increase in pH stability compared to free ACE. In order to fix receptor enzymes, the matrix should have an open structure to prevent interactions between the immobilized enzymes and acceptor molecules [19]. As cyanogen bromide (CNBr) reacts with hydroxyl groups on Sepharose 4B, it forms cyanate esters or imidocarbonates. These groups react readily with primary amines in very mild conditions, resulting in the covalent coupling of ACE to the Sepharose 4B [20]. The coupling procedure is relatively simple and reproducible [21]. Therefore, CNBr-activated Sepharose 4B was shown to be an efficient support for ACE immobilization.

### 2.2. Affinity Purification and Identification of T. flavidus Peptides

**Figure 2:**
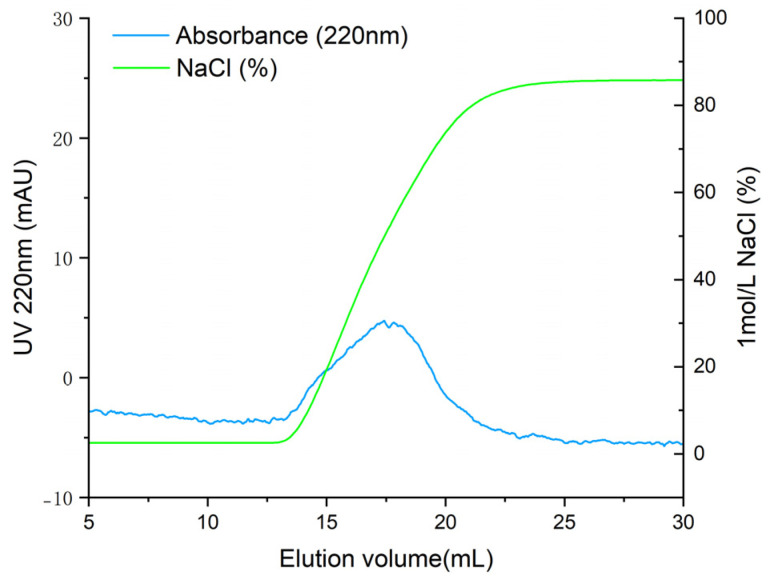
Elution profile of ACE-inhibitory peptides by ACE–Sepharose 4B affinity chromatography.

*T. flavidus* peptides were purified by affinity adsorption to the ACE–Sepharose 4B column. After reaching the adsorption equilibrium, elution was performed with a linear NaCl gradient (0–1 mol·L^−1^). As shown in Figure 2, the chromatogram of Sepharose 4B without ACE did not show an absorption peak, indicating that active groups of CNBr-activated Sepharose 4B were effectively blocked with glycine. One main elution peak was obtained on the elution curve of ACE–Sepharose 4B with the same experimental manipulation. The eluate was desalted and concentrated and subsequently sequenced by mass spectrometry. As shown in Table 1, 24 peptides with ALC ≥ 80% were identified. These peptides had lengths ranging from 4 to 12 amino acids, with molecular weights ranging from 358.2 to 1207.6 Da.

In affinity chromatography, biomolecules are separated by highly specific interactions between a target molecule and a ligand attached to a chromatography column [22]. Affinity chromatography is highly efficient and specific and become a potentially attractive approach for purification. Using immobilized metal affinity chromatography (IMAC-Ni^2+^), Sun et al. isolated a novel ACE-inhibitory peptide (Arg-Tyr-Arg-Pro) from lizard fish and demonstrated that IMAC may be useful for ACE inhibitor research [23]. According to Lu et al., lizard fish protein hydrolysates were purified by metal affinity-immobilized magnetic liposome (MA-IML), and an ACE-inhibitory peptide with an IC_50_ value of 108 µmol/L was identified as VYP [24]. In the study by Liu et al., a magnetic immobilized metal affinity chromatography matrix modified by poly (ethylene glycol) methyl ether (IMACM@mPEG) was prepared and applied for the rapid purification of ACE-inhibitory peptides from casein hydrolysate. A novel peptide with moderate ACE-inhibitory activity (IC_50_ value of 274 mmol·L^−1^) was identified as LYQEPVLGPVR [25]. Our study further confirms the feasibility and high effectiveness of affinity chromatography in the preparation of ACE inhibitory peptides from enzymatic hydrolysates. It indicated that ACE–Sepharose 4B is an effective method for the preparation of an effective method for purification of ACE-inhibitory peptide from *T. flavidus*.

### 2.3. The ACE-Inhibitory Activity Assay of Synthetic Peptides

**Figure 3:**
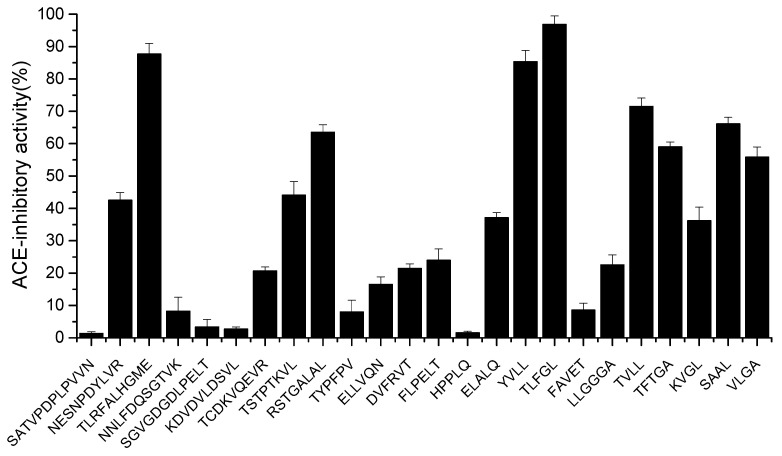
The ACE-inhibitory activity of synthetic peptides.

The peptides were chemically synthesized and validated for their ACE-inhibitory activity in vitro. As shown in Figure 3, twenty-four peptides (1 mg/mL) showed ACE-inhibitory activities ranging from 1.3 to 96.82%. Eight peptides (TLRFALHGME, RSTGALAL, YVLL, TLFGL, TVLL, TFTGA, SAAL, VLGA) with activity greater than 50% inhibition were further selected for IC50 determination. As shown in Table 2, the calculated IC50 values ranged from 57.5 to 5837.5 µmol·mL^−1^. TLFGL exhibited the highest ACE-inhibitory activity (IC50 = 57.5 µmol·mL^−1^), followed by TLRFALHGME and YVLL, while their IC50 values were 93.5 and 153.8 µmoL·L^−1^, respectively. However, TLFGL contains multiple hydrophobic amino acids, resulting in low solubility. Therefore, TLRFALHGME was selected for the next analysis.

The purified peptides with different binding affinities exhibited varied ACE-inhibitory activities. These differences might be related to binding sites of ACE, sequence length, and amino acid constitution [26]. It has been reported that the binding of the peptides to the enzyme active center of ACE played a critical role in the ACE-inhibitory activity [27]. However, the inhibition mechanism of TLRFALHGME will be further explored.

### 2.4. Inhibitory Kinetics Study

**Figure 4:**
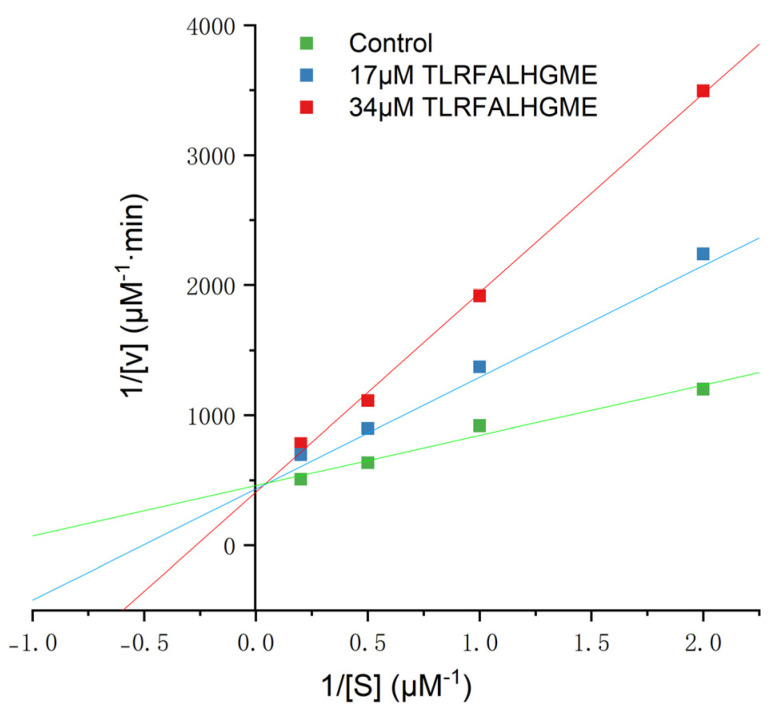
The Lineweaver–Burk plots of the reactions of ACE in the presence of TLRFALHGME. [S] = hippuryl-l-histidyl-l-leucine concentration; V = velocity of the reaction.

It has been reported that ACE-inhibitory peptides inhibit in different ways, including competitively, noncompetitively, and mixed-competitively [28]. Inhibition kinetics of TLRFALHGME were studied using Lineweaver–Burk plots. As shown in Figure 4, the slope changed while the y-intercept of 1/V remained unchanged for TLRFALHGME, suggesting that TLRFALHGME served as a competitive inhibitor by competing for the binding site on ACE. Kinetic studies indicated that TLRFALHGME possessed a competitive inhibition mode. 

### 2.5. Molecular Docking Simulation

**Figure 5:**
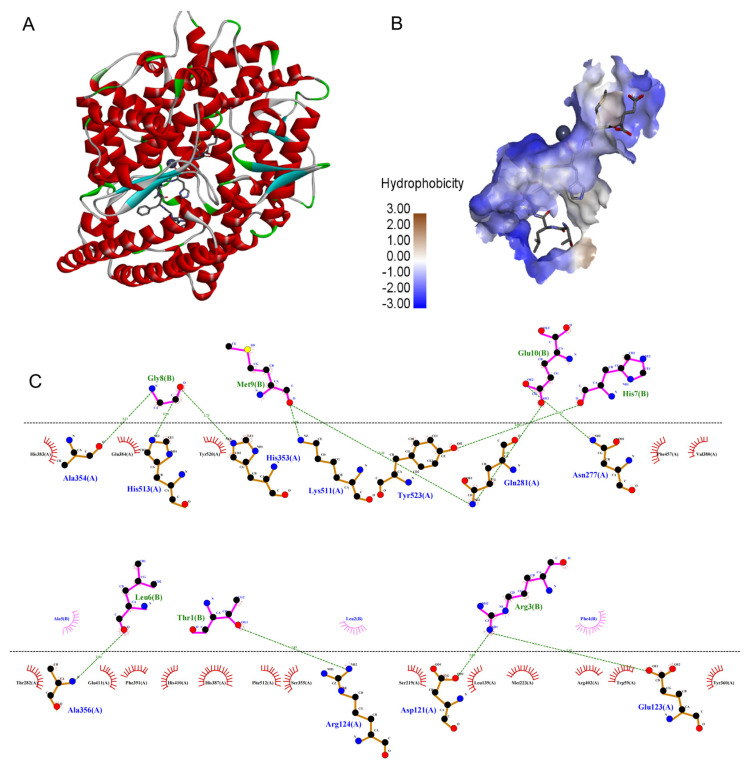
The molecular docking simulations of TLRFALHGME with ACE (PDB: 1O8A): (**A**) general overview and (**B**) the best-ranked docking pose of TLRFALHGME; (**C**) the interactions between TLRFALHGME and the residues of ACE.

In recent years, molecular docking simulations have become an important tool for understanding the interaction mode and the structure-activity relationships of ligands with receptors [29]. As shown in Figure 5, TLRFALHGME bonded to the enzyme active center and occupied the substrate-binding channel of ACE. The interaction of TLRFALHGME with ACE involves hydrogen bonds, hydrophobic interactions, and van der Waals forces. TLRFALHGME formed twelve hydrogen bonds with the residues of the ACE active site (Ala354, His513, His353, Lys511, Tyr523, Gln281, Asn277, Ala356, Arg124, Asp121, and Glu123). Moreover, TLRFALHGME showed hydrophobic contacts with Glu384, His 383, Tyr 520, Phe 457, Val380, Thr282, Glu411, Phe391, His410, His 387, Phe512, Ser355, Ser219, Leu139, Met223, Arg402, Trp59, and Tyr360.

Previous studies have shown that there were at least three active site pockets in ACE: S1 pocket (Gln281, His353, Lys511, and His513), S2 pocket (Ala354, Glu384, and Tyr523), and S1’pocket (Glu162) [30]. In our study, TLRFALHGME formed four hydrogen bonds with the S1 pocket (Gln281, His353, Lys511, and His513) and two hydrogen bonds with the S2 pocket (Ala354 and Tyr523). TLRFALHGME also interacted with Glu384 residue in the S2 pocket through a hydrophobic interaction. According to the results, TLRFALHGME is effective in interacting with ACE at its active site and forming a stable complex, thus leading to the inhibition of enzyme activity. Overall, our results are consistent with the previous reports [31,32].

### 2.6. Molecular Dynamics Simulations

**Figure 6:**
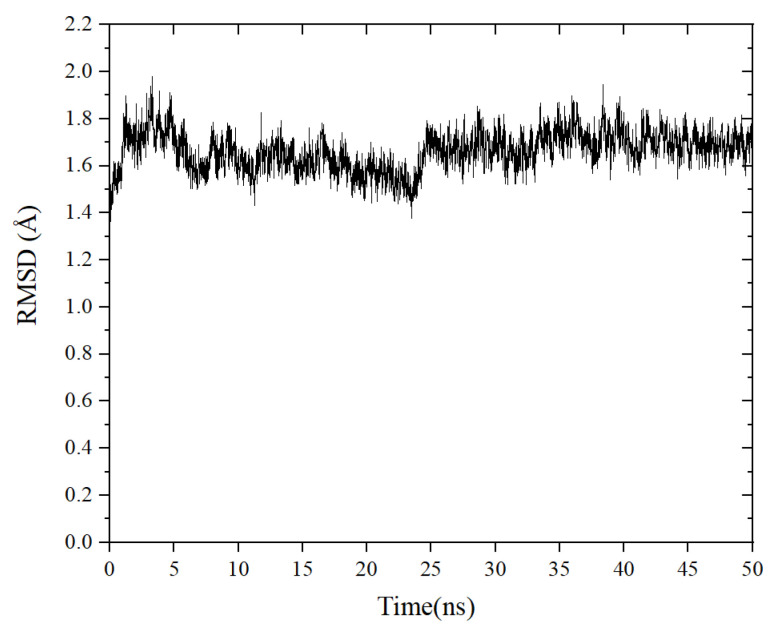
The change root mean square deviation (RMSD) of the ACE-TLRFALHGME complex over time.

MD simulations were carried out to explore the stability of the binding tendency of the ACE–peptide complex. RMSD is an important parameter for measuring the stability of the protein–ligand complex [33]. It can be used to describe the conformational deviations during the dynamic simulation, which can reflect the stability of the ACE–peptide complex [34]. As shown in Figure 6, the root mean square deviation (RMSD) value of the ACE-TLRFALHGME complex fluctuated sharply in the early stages of the simulation and stabilized at around 0.16 nm to 0.18 nm after 25 ns. The result of the binding free energy calculation (MM/PBSA) is shown in Table 3. The total binding energy ΔGbinding was calculated as −82.7 ± 9.3 kJ·mol^−1^, indicating the favorable binding of TLRFALHGME with ACE. The contribution of van der Waals energy and electrostatic energy to the total binding energy were −104.7 ± 6.6 kJ·mol^−1^ and −409.2 ± 35.1 kJ·mol^−1^. Our results indicated that TLRFALHGME could bind ACE rapidly, forming a highly stable ACE-TLRFALHGME complex. The van der Waals and electrostatic interactions contributed to the maintenance of the stability of ACE-TLRFALHGME complexes, which agreed with the molecular docking simulation. 

### 2.7. Surface Plasmon Resonance (SPR) Analysis

**Figure 7:**
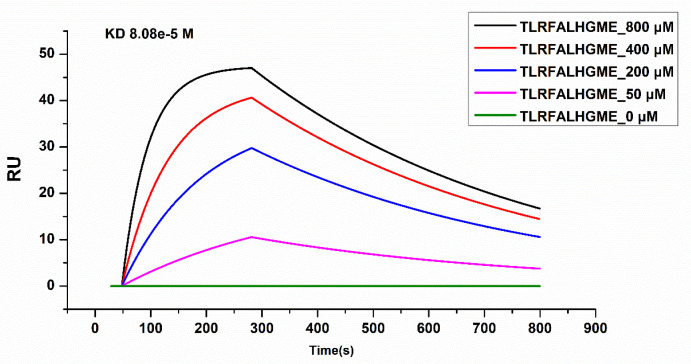
Surface plasmon resonance (SPR) analysis of interactions between TLRFALHGME and ACE.

SPR was used to analyze the physical interactions between TLRFALHGME and ACE. SPR is an optical technique based on the detection of SPR biosensor chips and is widely used to characterize molecular interactions [35]. The binding affinity of the ACE–peptide complex was evaluated by determination of the binding affinity (KD) using SPR [36]. ACE was coupled with a sensor chip, and TLRFALHGME was added onto the chip at 0, 50, 200, 400, and 800 µmol·L^−1^ (Figure 7). Analysis of the sensogram was conducted to determine association and dissociation rate constants: Ka = 0.247 M^−1^ s^−1^ and Kd = 0.002 s^−1^. The KD value of the ACE-TLRFALHGME complex calculated from its kinetic parameters (Kd/Ka) is 80.9 μmol·L^−1^.

According to SPR results, TLRFALHGME has a high binding affinity for ACE, which validates previous molecular simulation results. Due to the relatively long dissociation time, the kinetics of TLRFALHGME displayed the characteristics of a relatively slow kinetics; hence, it may have a slow release and sustained drug efficacy.

### 2.8. Antihypertensive Activity of TLRFALHGME

**Figure 8:**
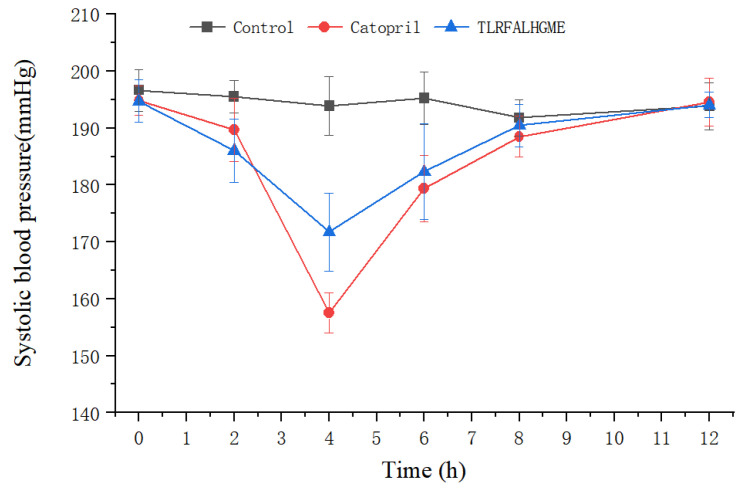
SBP changes in SHRs after the intravenous administration of TLRFALHGME.

After intravenous administration of TLRFALHGME, antihypertensive effects were evaluated on spontaneously hypertensive rats (SHR). The SHR is the most widely used animal model for studying human essential hypertension [37]. As shown in Figure 8, TLRFALHGME had good effects on lowering blood pressure. A significant reduction in SBP was observed between 2 and 8 h (*p* < 0.05), with the lowest SBP of 171 mmHg occurring around 4 h after TLRFALHGME was administered. The SBP then recovered to 190 mmHg after 8 h.

In recent years, a number of ACE-inhibitory peptides have been demonstrated to have an antihypertensive effect on SHR. Li et al. identified a novel ACE-inhibitory peptide CSBp5 from corn silk, which exhibited antihypertensive effects in SHRs via the inhibition of ACE [38]. Chen et al. showed that ACE-inhibitory peptide WGAP significantly reduced systolic and diastolic blood pressure in hypertensive rats by up to 42.66 ± 2.87 and 28.56 ± 2.71 mmHg [39]. In this study, we reported the effect of TLRFALHGME on lowering blood pressure. TLRFALHGME had great potential to be used as an antihypertension agent, which is comparable to the effectiveness of captopril treatment. TLRFALHGME could be a promising compound for further modification and optimization in order to improve its biological activity and pharmacokinetic properties [40].

## 3. Materials and Methods

### 3.1. Materials

*Takifugu flavidus* were purchased from Zhangzhou City of Fujian Province, China. The skin was shelled, husked, and used for further experimentation. Alcalase, pepsin, and trypsin were purchased from Sinopharm Group (Beijing, China); angiotensin-I-converting enzyme, hippuryl-l-histidyl-L-leucine (HHL), hippuric acid, and CNBr-activated Sepharose 4B were purchased from Sigma-Aldrich (St. Louis, MO, USA). Captopril was procured from MedChem Express (Monmouth Junction, NJ, USA). All other chemicals/reagents used were of analytical grade or HPLC grade.

### 3.2. Immobilization ACE onto CNBr-Activated Sepharose 4B

ACE was bonded to CNBr-activated Sepharose 4B according to procedures previously described with slight modifications (Homaei, 2015). A total of 5 U ACE was dissolved in 0.1 mol·L^−1^ NaHCO_3_ buffer containing 0.5 mol·L^−1^ NaCl, pH 8.3–8.5. CNBr-activated Sepharose 4B was washed using a cold solution of 1 mM HCl. The gel was then filtered and washed with distilled water and 0.1 mol·L^−1^ NaHCO_3_ (0.5 mol·L^−1^ NaCl, pH 8.3) successively. The filtrate was transferred to ACE solution and gently stirred overnight at 4 °C. The unreacted ACE was washed away with 0.1 mol·L^−1^ NaHCO_3_ (0.5 mol·L^−1^ NaCl, pH 8.3). The remaining active groups were blocked with 0.2 mol·L^−1^ glycine (pH 8.0) for 2 h at room temperature. The suspension was washed three times with 0.1 mol·L^−1^ sodium acetate (0.50 mol·L^−1^ NaCl, pH 4.0), followed by 0.1 mol·L^−1^ Tris-HCl (0.5 mol·L^−1^ NaCl, pH 8.0). The matrices were then packed into 5 mL polypropylene columns (Qiagen, Germany) and named ACE-MB affinity columns.

### 3.3. Preparation of Peptide from T. flavidus

The peptide was prepared according to previously published methods [18]. The skins of *T. flavidus* were hydrolyzed by alcalase (2000 U·g^−1^) for 5 h under the optimum temperature and pH conditions (55 °C, pH 8.0). The *T. flavidus* hydrolysates were filtered using a continuous flow ultrafilter (STAR Biotechnology Co., Ltd., Xiamen, China) with ultrafiltration membranes (MWs < 1 kDa). Fractions with MWs < 1 kDa were collected and lyophilized.

### 3.4. Affinity Purification of ACE-Inhibitory Peptides

The antihypertensive peptide was dissolved in Tris-HCl (50 mmol·L^−1^, pH 7.4) and loaded onto immobilization ACE affinity column pre-equilibrated with 50 mmol·L^−1^ Tris-HCl buffer (pH 7.4). After loading, the column was washed with equilibration buffer (50 mmol·L^−1^ Tris-HCl, pH 7.4) until the absorbance at 220 nm returned to a stable baseline. The elution was performed using Tris-HCl (50 mmol·L^−1^, pH 7.4) with 1 mol·L^−1^ of NaCl at a flow rate of 0.2 mL·min^−1^. The salt elute with UV_220_ absorbance peaks were collected, desalted, and lyophilized for the next experiments.

### 3.5. Analysis of the Amino Acid Sequence by UPLC-MS/MS

Q Exactive mass spectrometer (Thermo Fisher, Waltham, MA, USA) was used to analyze affinity chromatographic elute components [41]. After desalination, the sample was loaded onto an Acclaim PepMap C18 column (Acclaim PepMap RPLC C_18_, 75 μm i.d. × 150 mm, 3 μm). The mobile phase A contained 2% acetonitrile (with 0.1% formic acid, *v*/*v*), while the mobile phase B contained 80% acetonitrile (with 0.1% formic acid). Gradient elution was carried out with a gradient of 6–95% B, with a flow rate of 300 nL·min^−1^. The MS data were then processed with PEAKS Studio using de novo sequencing (Bioinformatics Solutions Inc., Waterloo, QC, Canada). The peptides were selected with a confidence score of ALC > 80%.

### 3.6. Screening for ACE-Inhibitory Peptides

The peptides were prepared by the fmoc solid-phase method (purity ≥ 98% by HPLC) at Genscript Biotech Corporation (Nanjing, China). ACE-inhibitory activity of the synthesized peptides was evaluated according to the method of Ma. et al., with some modifications [27]. In brief, 50 µL of sample was added into 150 µL of 5 mmol·L^−1^ hippuryl-l-histidyl-l-leucine (HHL) and incubated for 10 min at 37 °C. Then, the enzymatic reaction was initiated by addition of 50 µL of 50 mU/mL ACE solution and kept at a constant temperature of 37 °C for 45 min. The reaction was then terminated by adding 250 µL of 1 mol·L^−1^ HCl. RP-HPLC (Waters, Milford, MA, USA) was used to measure hippuric acid (HA) formation at 220 nm. Content of reaction products hippuric acid (HA) was determined using RP-HPLC (Waters, Milford, MA, USA) at 220 nm. The IC_50_ value represents the concentration of the peptide that inhibits 50% of ACE activity under the assay conditions.

### 3.7. Inhibitory Kinetics Study

The inhibitory kinetics of TLRFALHGME were analyzed using Lineweaver–Burk plots according to the protocol described by Lin et al. [42]. The reaction mixtures consisted of 100 μL of HHL (0.1, 0.5, 1, 2, and 5 mmol·L^−1^) as a substrate, 50 μL of 5 mmol·L^−1^ ACE, and 50 μL of each sample solution (10, 20, 40 μg·mL^−1^ of TLRFALHGME) were assayed in the same conditions as Method 3.6. The Lineweaver–Burk plot was drawn based on reciprocal of initial reaction rate (1/V) against the substrate concentration (1/[S]), and the Km and Vmax were calculated.

### 3.8. Molecular Simulations

Discovery Studio 2019 (NeoTrident Technology, Beijing, China) was employed to analyze the binding affinities and modes of interaction between ACE and peptide [43]. The molecular structures of TLRFALHGME were drawn with ChemDraw Pro 16.0 software.

The 3D coordinates of the structure of human ACE (PDB: 108A) were downloaded from the PDB (https://www.rcsb.org/structure/1O8A (accessed on 15 August 2023)). Molecular and protein files were converted into PDBQT format, where water molecules were excluded, and polar hydrogen atoms were added for docking analysis. Molecular docking results were selected based on their docking scores and binding energies. According to docking scores and binding energies, the best docking result was selected.

MD simulations were conducted on the Yinfo Cloud Computing Platform (CCP) using AmberTools 20 (https://cloud.yinfotek.com/ (accessed on 20 August 2023)) [44]. The ACE-TLRFALHGME complex was simulated using the Amber ff14SB all-atom force field. The complex was solvated in TIP3P water molecules within a dodecahedron box, ensuring a minimum distance of 1.5 nm between the protein and the box’s edge. The simulation consisted of two stages of energy minimization, followed by heating, equilibration, and production. A total of 50 ns simulation time was conducted, with the molecular dynamic parameters set according to the reference [45]. The binding-free energies (ΔGbind) were estimated using MM-GBSA and MM-PBSA procedures in AMBER12 [46]. The following equation was used:

(1) $$∆G_{bind}=∆G_{complex}-(∆G_{receptor}-∆G_{ligand})$$

### 3.9. Surface Plasmon Resonance (SPR) Assay

The affinity interaction between TLRFALHGME and ACE was verified by SPR experiments [47]. In brief, 5 ug ACE was fixed on a COOH sensor chip by capture-coupling. Afterwards, 50–800 μmol·L^−1^ of TLRFALHGME were injected sequentially into the chamber in PBS running buffer. The SPR assay was performed at 25 °C on an OpenSPRTM (Nicoya Lifesciences, Waterloo, QC, Canada). The conditions for the analysis were set at a flow rate of 20 μL·min^−1^, the binding time was 240 s, and the disassociation time was 480 s. Chips were regenerated using a regeneration buffer (0.25% SDS). A one-to-one diffusion-corrected model was fitted to the wavelength shifts corresponding to the series of TLRFALHGME concentrations. The kinetic constant analysis was performed using TraceDrawer 1.9.1 software (Ridgeview Instruments AB, Uppsala, Sweden) to determine the association constant (Ka) and dissociation constant (Kd). The affinity constant (KD) was calculated as the ratio kd/ka.

### 3.10. Antihypertensive Effect In Vivo

Male SHRs (10 weeks, 220 ± 20 g body weight) were purchased from Vital River Laboratory Animal Technology Co., Ltd. (Beijing, China). Following an acclimation period of one week, SHRs with systolic blood pressure (SBP) higher than 180 mmHg were randomly divided into groups of ten rats. Animals were housed at 25 °C under a light–dark cycle of 12 h/12 h. TLRFALHGME was injected via tail vein at a dose of 4 mg/kg body weight.

Under the same conditions, a saline group was used as the negative control and captopril (5 mg·kg^−1^) as the positive control. The SBP of all rats was measured using a BP-98A blood pressure monitor (Softron Biotechnology, Beijing, China) before and after intravenous administration. The rats were all treated humanely according to the guidelines of the National Institutes of Health and Use of Laboratory Animals and approved by the Ethics Committee of Guangdong Medical Laboratory Animal Center (no. 20211001, approved on 9 September 2021).

### 3.11. Statistical Analysis

Data were analyzed by one-way ANOVA (Analysis of Variance), and mean comparisons were carried out by Duncan’s multiple range test using SPSS statistics software v.20.0 (IBM SPSS Inc., Chicago, IL, USA).

## 4. Conclusions

In this study, an affinity chromatography of Sepharose-immobilized ACE was applied for the purification of ACE-inhibitory peptides from *T. flavidus*. In total, 24 peptide sequences of eluted fractions were identified by LC-MS/MS and subjected to ACE-inhibitory activity assay. One potential ACE-inhibitory peptide (TLRFALHGME) possessed the highest ACE-inhibitory activities with IC_50_ values of 93.5 µmol·mL^−1^. Kinetic studies indicated that TLRFALHGME possessed a competitive inhibition mode. The results of molecular docking, molecular dynamics, and SPR analysis confirmed the direct interaction between the peptide and ACE. This is also in contrast to our previous work, in which we purified a novel ACE-inhibitory peptide PPLLFAAL using semi-preparative RP-HPLC, sephadex G-15 gel chromatography, and RP-HPLC [18]. The affinity method of Sepharose-immobilized ACE has the advantages of specificity and efficiency, which can be widely applied to the purification of potentially ACE-inhibitory peptides from natural sources.

## Figures and Tables

**Table 1:** Peptide sequences of elution fractions identified by LC-MS/MS analysis.

NO	Peptides Sequence	Length	Mass (Da)
1	SATVPDPLPVVN	12	1207.6448
2	NESNPDYLVR	10	1205.5676
3	TLRFALHGME	10	1173.5964
4	NNLFDQSGTVK	11	1221.5989
5	SGVGDGDLPELT	12	1158.5405
6	KDVDVLDSVL	10	1101.5918
7	TCDKVQEVR	9	1076.5283
8	TSTPTKVL	8	845.4858
9	RSTGALAL	8	787.4552
10	TYPFPV	6	722.3639
11	ELLVQN	6	714.3912
12	DVFRVT	6	735.3915
13	FLPELT	6	718.3901
14	HPPLQ	5	590.3176
15	ELALQ	5	572.317
16	YVLL	4	506.3104
17	TLFGL	5	549.3162
18	FAVET	5	565.2748
19	LLGGGA	6	486.2802
20	TVLL	4	444.2948
21	TFTGA	5	495.2329
22	KVGL	4	415.2794
23	SAAL	4	360.2009
24	VLGA	4	358.2216

**Table 2:** The ACE-inhibitory activity of the top 8 peptides.

NO	Peptides Sequence	IC_50_ (µmol/L)
1	TLFGL	57.5
2	TLRFALHGME	93.5
3	YVLL	153.8
4	TVLL	582.1
5	SAAL	882.9
6	TFTGA	694.3
7	VLGA	1372.9
8	RSTGALAL	5837.5

**Table 3:** Predicted binding free energies of ACE-TLRFALHGME complexes.

Complex	Average	Std. Dev.	Std. Err. of Mean
ΔEvdw	−104.7043	6.6073	1.2958
ΔEelec	−409.2481	35.1386	6.8913
ΔGpolar	446.5627	28.3400	5.5579
ΔGnonpolar	−15.3484	0.3851	0.0755
ΔGbinding	−82.7382	9.2771	1.8194

Note: ΔEvdW, ΔEelec, ΔGpolar, and ΔGnonpolar are binding energy components of van der Waals, electrostatic, polar, and nonpolar solvation energies, respectively.

## Data Availability

The data presented in this study are available on request from the corresponding author.
